# Case Report: Lateral C1–C2 puncture for intrathecal baclofen therapy: an alternative effective and safe approach after spinal cord injury

**DOI:** 10.3389/fpain.2025.1571716

**Published:** 2025-06-10

**Authors:** Rayan Fawaz, Hayat Belaid, Baptiste Eklu, Jean Baptiste Thiebaut, Manon Duraffourg

**Affiliations:** ^1^ Department of Neurosurgery, Hôpital de la Fondation Adolphe de Rothschild, Paris, Cedex, France; ^2^ Unité de Neuromodulation Polyvalente, Service de Neurochirurgie Fonctionnelle, Hôpital Neurologique et Neurochirurgical Pierre Wertheimer, Hospices Civils de Lyon, Lyon, France; ^3^ CETD, Hôpital Neurologique Pierre Wertheimer, HCL, Bron, France; ^4^ Service de Médecine Physique et de Réadaptation, Hôpital Henry Gabrielle, Hospices Civils de Lyon, Lyon, France

**Keywords:** C1–C2 puncture, cervical intrathecal baclofen, spinal cord injury, intrathecal, pump

## Abstract

Spasticity is a neurological disorder that disrupts the regulation of muscle tone following an injury to the central nervous system, such as spinal cord injury. Baclofen is the most effective medication for treating spasticity and can be delivered via a pump connected to an intrathecal catheter. The catheter is typically inserted via a lumbar punction and advanced up to the level corresponding to the disturbing spasticity. But this may not be possible, especially when cervical level is involved. We present the case of a patient with severe spasticity after a traumatic cervical spinal cord injury who successfully underwent a lateral C1–2 puncture for placement of a retrograde catheter to the C4 level, after an unsuccessful attempt at catheter placement via a lumbar puncture. The patient experienced a significant reduction in spasticity with no reported worsening during the 8 months follow-up period. The catheter placement via a lateral C1–2 puncture guided by innovative imagery with 3D reconstruction, may serve as an effective and safe alternative to deliver baclofen at the cervical level. Relevance of cervical ITB is discussed and issues involved are considered. The mechanism of action of ITB at cervical level, which is far from fully clarified, is crucial to reach the best clinical outcome and avoid si de effects and complications. Few clinical cases were published; hence the importance to present this case.

## Introduction

Spasticity is a neurological disorder that disrupts the regulation of muscle tone following an injury to the central nervous system, such as spinal cord injury (SCI).

Baclofen is the most effective medication for treating spasticity and can be delivered intrathecally via a pump connected to a catheter (1). It is now well established that the diffusion of intrathecal medications is limited. The position of the tip of the catheter is an essential factor for success (2–4). This catheter is typically inserted via a lumbar punction (LP) and advanced up to the level corresponding to the disturbing spasticity. If the catheter placement via lumbar puncture is unsuccessful, catheter placement via a lateral C1–2 approach might serve as an alternative because a percutaneous puncture is achievable at this level (5). This approach, developed to perform cordotomies and afterwards cervical myelographies, benefited from innovative imagery with 3D reconstruction in the operating room (6).

We present the case of a patient with severe spasticity secondary to a traumatic cervical SCI and successfully treated by cervical intrathecal baclofen (ITB) via a catheter introduced by a lateral C1–C2 puncture and advanced in a retrograde direction down to the cervical level after one unsuccessful attempt through a lumbar puncture. The varied events that occurred during the complex management of the patient's spasticity give us the opportunity to discuss different set of issues which are not often addressed on the importance of the catheter tip placement and the benefits of the cervical ITB.

## Case report

A 32-year-old patient underwent surgery in November 2022 following an ASIA A SCI at the C4–C5 level due to a paragliding traumatic accident. An emergency anterior decompression with C4–C5 arthrodesis was performed. In the postoperative period, the patient developed severe spasticity and spasms in all four limbs which could cause falls, with a Modified Ashworth Scale (mAS) score of 4/4 and a Penn Spasm Frequency Scale score of 4, both resistant to oral baclofen at a dose of 120 mg per day administered in three divided doses.

The patient provided signed informed consent to participate in a study. In March 2023, a programmable Synchromed™ II 20 ml device (Medtronic Inc, MN) connected to an intrathecal catheter advanced up to the C4 level was implanted via a lumbar puncture for ITB infusion, with a continuous dose of 100 mcg/24 h. The patient showed significant improvement in spasticity, with a modified Ashworth score (mAS) 0/4.

Three weeks later, the patient developed a meningocele accompanied by lumbar scar dehiscence, which required removal of the device and initiation of probabilistic antibiotic therapy.

After wound healing and completion of antibiotic treatment, faced to spasticity recurrence, the patient underwent another intrathecal pump implantation via lumbar puncture. However, during the procedure, the catheter could not be positioned at the cervical level and was instead placed at the T2 level. As a result, spasticity remained unchanged with a mAS always at 4/4 using the baclofen equivalent previous continuous dose.

Finally, a lateral C1–2 approach was proposed to optimally position the catheter at the cervical level. The preoperative evaluation included an angio CT scan and a spinal MRI which did not reveal any anatomical variations in the vertebral artery trajectory, nor any cranio cervical or spinal cord abnormalities. A simulated puncture was performed, and the distance skin-rachis measured.

**Figure 1:**
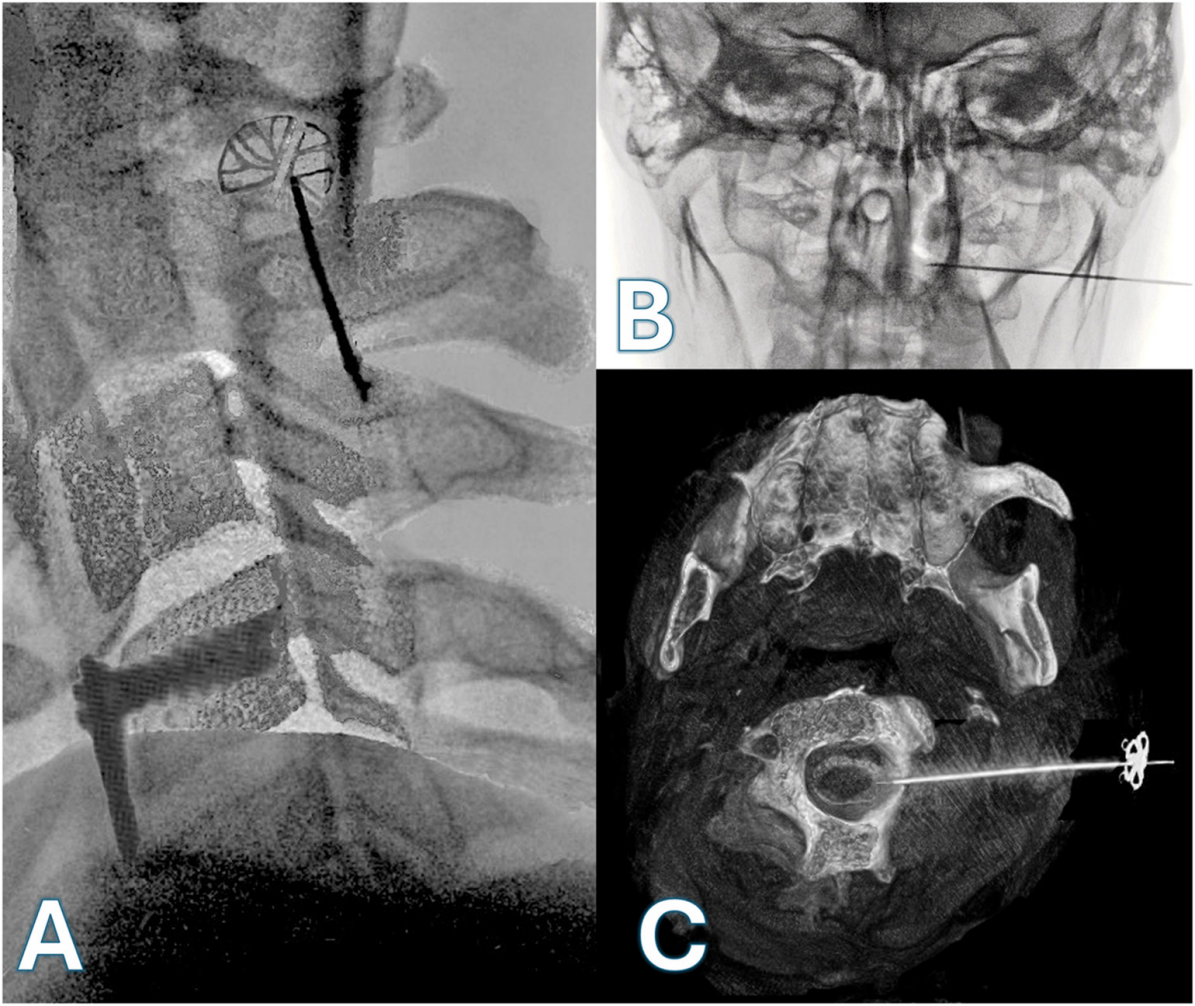
Fluoroscopic and CT-guided imaging of C1–C2 intrathecal access. **(A)**: Lateral fluoroscopic view showing the trajectory of the Tuohy needle through the C1–C2 interlaminar space toward the intrathecal compartment. **(B)**: Anteroposterior fluoroscopic view confirming midline alignment of the needle. **(C)**: Axial CT image following contrast injection, clearly outlining the spinal cord and confirming intrathecal placement.

The procedure was performed under general anesthesia in the operating room, using a 3D imaging system, the O-Arm (Medtronic Navigation Inc.). The patient was positioned supine with the head secured in a radiolucent Mayfield horseshoe head holder, ensuring a neutral position. A Metrix* plate served as a marker, fixed with a transparent drape to guide the puncture. The plate was positioned on the lateral cervical area approximately 1.5 cm below and behind the mastoid process (Figure 1A).

**Figure 2:**
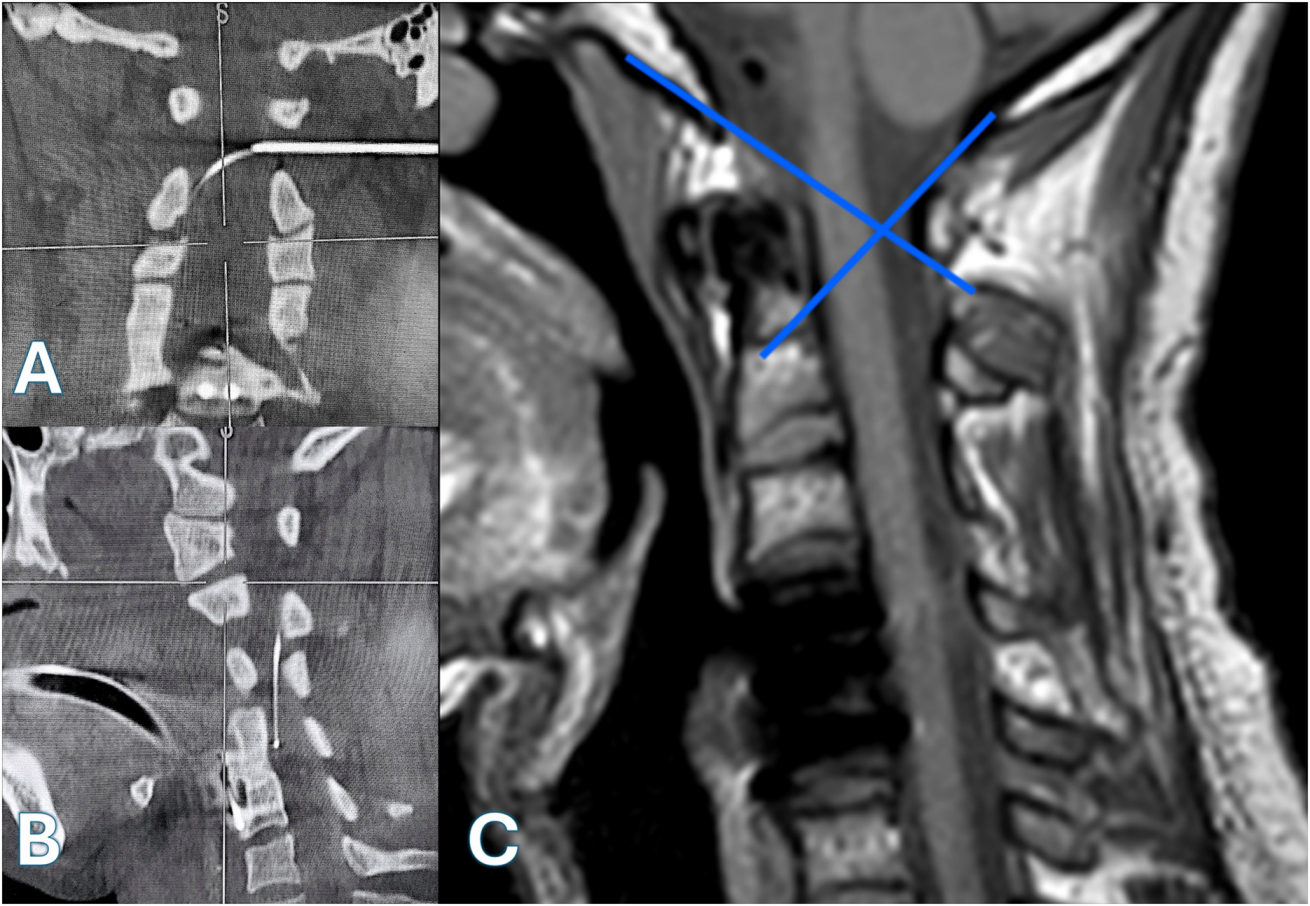
[**(A)**—coronal view, **(B)**—sagittal view]: intraoperative CT scans with multiplanar reconstructions demonstrating precise placement of a Tuohy needle at the C1–C2 interlaminar space under 3D image guidance, with the catheter tip positioned at the C4 level. **(C)** Preoperative sagittal T1-weighted MRI illustrating the anatomical modeling of the “Flair Point”.

Initial lateral fluoroscopy was used to guide a Tuohy needle toward the posterior spinal canal, just behind the Flair Point (FP) (Figure 2C), which corresponds to the posterior boundary of the spinal cord between C1 and C2, slightly within the spinolaminar line. After advancing the needle 4–5 cm, fluoroscopy helped to guide the needle tip toward the target (Figure 1B). When a resistance was felt at the pre op planned depth, the needle direction is controlled on the axial reconstruction of a 3D acquisition. Then the needle is pushed with a sharp movement to go through the yellow ligament and the duramater. The stylet was withdrawn to check for cerebrospinal fluid (CSF) return. A myelography is carefully performed, starting by injecting 1 ml and checking the contrast pulsation by fluoroscopy before injecting 5 cc of intrathecal contrast (Iopamiron*). A 3D imaging (Figure 1C) is performed. After confirming the needle's position relative to the spinal cord in the posterior citern, the intrathecal catheter (Ascenda*, Medtronic, Inc.) is introduced and advanced down to the C4 level (Figures 2A,B). A skin incision centered at the needle site was made. The guidewire and the needle were removed, taking care not to move the catheter which was anchored. The previous pump was then removed through an abdominal incision and the intrathecal catheter tunneled from the retromastoid incision to the abdominal pump pocket over the thoracic cage using a shunt passer. The intrathecal catheter was connected to the pump.

No complications related to the C1–2 puncture were observed. Additionally, no wound or catheter-related issues were noted after 8 months follow-up. The patient quickly experienced a significant reduction in spasticity that the baclofen daily dose need to be reduced. No reported cases of spasticity worsening were reported during the 8 months follow-up period with a mAS score of 1/4 and a Penn Spasm Frequency Scale score of 1. The pump baclofen concentration was changed to 2,000 µg/m with increasing the dose to 514 µg/24 h. The patient was very satisfied by the results. The disappearance of the violent muscle spasms, the primary goal, allowed to intensify rehabilitation.

## Discussion

After three decades of clinical use, ITB is a well-established therapy proven effective in treating refractory spasticity in SCI. It acts as a centrally acting gamma-aminobutyric acid (GABA)-B agonist, functioning as a muscle relaxant by reducing reflex transmission at the spinal cord level (1).

The case described in this study highlights the challenges clinicians may face in achieving optimal catheter placement after SCI (2–4).

First, positioning the catheter adjacent to the cervical lesion was achieved successfully few months after the trauma, but the cervical positioning will no longer be possible one year later.

At that point, the treatment proved ineffective with a thoracic positioning, underscoring the limited diffusion of intrathecal drugs. Optimal therapeutic effect requires that the catheter tip be precisely positioned at the appropriate spinal level. In several situations, the catheter cannot be positioned correctly after lumbar puncture (5).

A lateral C1–2 approach was chosen to access the intrathecal space for placement of a retrograde catheter to the C4 level because it's a direct one due to the lack of obstacles in the needle's path. The space between C1 and C2 was free, with no articular processes. The retromedullary cistern was also relatively spacious. Additionally, no vessels were observed in the trajectory of the needle (6).

It is a safe procedure with minimal risks with the advancements in imaging with the Cone beam CT (O'arm*), including the use of bi-plane x-rays and 3D reconstructions in the operating room, which enables real-time monitoring and confirmation of the needle's position intraoperatively (6, 7). It is crucial to localize the dorsal margin of the spinal cord on a side view. We consider intra-dural contrast injection as an added safety measure although a bit risky, but it is not mandatory (8–10). Peckham introduced an osseous landmark, the “Flair Point” (FP), that closely approximated the dorsal margin of the spinal cord. The FP represents the triangular “flaring” of the posterior C1 arch at its junction with the anterior arch, where the vertebral artery enters the spinal canal (11).

The best target to ensure safe and accurate needle positioning in the CSF posterior cistern is located in the posterior third of the spinal canal between the FP and the spinolaminar line at the upper mid C1–2 interspace. It is important to avoid targeting too far below, where the C2 nerve root is located, or too far posteriorly where the dural sheath is narrow, to prevent contact with the posterior epidural veins, too far anteriorly to prevent contact with the posterior spinal cord pia mater (8–10).

### Indications

The C1–2 intrathecal catheter implantation with a classical technique has been previously described for spasticity and dystonia in patients with cerebral palsy sometimes associated to kyphoscoliosis and multilevel arthrodesis (5, 12, 13). The method was also proposed to treat pain, in most cases cranio cervical cancer pain (14, 15). No complications have been reported, but the number of cases is small.

### Contraindications

We saw earlier that contraindications to the C1–2 lateral approach are identified by pre-implant assessment with a cervical angio CT scan and a MRI (8–10).

### Complications

If the described method is strictly applied, the complication rate related to the procedure is virtually non-existent (8–10). Only the myelography involves a certain amount of risk (6). Furthermore, the cervical ITB is well tolerated, no hypoventilation was described at the usual dosages. However, potential devices complications, concerning mainly the catheter and errors in pump refill procedure, are very well known and had been extensively discussed (16–18).

### Alternatives

Various methods were proposed to replace the catheter tip at the cervical level when the lumbar approach is challenging. Lateral percutaneous C1–C2 approach seemed to us the least aggressive with minimal risks. Percutaneous cervical posterior puncture are hazardous as the spinal cord is directly in the puncture line (19–22). Open surgical procedures or a trans ventricular method are more aggressive (23, 24).

### Rational

The ability to manage spasticity is excellent with current clinical practices. Catheter tips are most commonly placed in the thoracic spine. To treatment efficacy and safety, it is important to understand the impact of changing the usual parameters like the catheter tip placement when an alternative may be necessary. Our case report is a good example. ITB was effective on two occasions when the catheter tip was positioned at the cervical level and ineffective at the thoracic level, even by greatly increasing baclofen doses. The impact of the catheter tip level is now well established (3, 4, 25, 26). Fundamental studies demonstrated that the effect of a drug injected into the CSF is limited. Without to go into details, it is essential to be aware of some physiological data.

Baclofen acts at the level of spinal cord receptors. Its spinal cord concentration depends of the CSF concentration at the same level and of its solubility in the nerve tissue. Despite its low hydro solubility, the majority of injected baclofen crosses the nerve bar near the point of infusion and its concentration falls drastically farther away. The infused drug concentration depends on the injected amount and the CSF dissemination. CSF flow is the crucial factor of the drug dissemination with the specific drug properties like its buoyancy. Drug diffusion is very low, its effect is considered negligible. CSF flow is not a continuous mixing one caudad and cephalad which was once portrayed. CSF flow oscillates along a craniocaudal axis. Several forces such as cardiac motion (the most important), respiration, CSF turnover and body movement contribute to the CSF pulsatile flow (4, 27–30).

He flow velocity is greatest in the cervical spine and is essentially absent in the distal lumbar sac. This results in a greatest exposure to the cervical spinal cord when the catheter tip is located at the high cervical level with a lower concentration gradient over a larger spinal area in the cervical spine in comparison with the catheter at the lumbar region (27–30). The structures in contact with the CSF (bones, roots) can modify CSF flow by creating vortices and this may lead to rapid changes of drug movements. Benchtop and in silico modeling give a representation of these phenomena, but this cannot yet be effectively implemented in clinical practice (4, 29, 30). To date, there have been no studies to predict the best catheter tip placement to treatment efficacy and safety, to understand the clinical implications of the catheter direction upward or downward with retrograde infusion, hence the importance to present clinical cases results (4).

Furthermore, when utilizing intrathecal drug delivery via an implantable programmable infusion system, multiple factors such as concentration, volume, and rate of infusion can contribute to the overall distribution. The ability of a large volume IT bolus injection has been shown to facilitate improved distribution to the neurons of the spinal cord (31). But the practical interest is limited, because lower volume infusion by higher baclofen concentration allows to limit the refilling frequency even if the therapeutic effect is less.

### ITB indications

ITB is an effective and safe option for the treatment of spasticity refractory to conventional therapies, pharmacotherapy (orally administered baclofen) or rehabilitation (to control spasms triggers) (32). Studies comparing clinical outcomes of long-term intrathecal vs. oral baclofen use demonstrated significantly lower levels of spasm frequency and severity associated with intrathecal baclofen treatment (33–35). A further advantage is that ITB can be individually regulated to allow precise adjustment of the dose that can vary over a 24 h period. This makes it possible to avoid a drop in available strength when residual motor control is essential to maintain functional activities.

We have had experience of ITB since 1987. We have studied the long-term efficacy and functional benefits of ITB for severe spinal spasticity which, for tetraplegic patients, depends very much on the personal clinical situation (36–38). Patient pathway and ITB indication were determined according to a multidisciplinary approach involving rehabilitation physicians and neurosurgeons and to the guidelines published by medical associations (1, 39, 40). We did not perform an initial percutaneous bolus test which would not have allowed to predict the result of the cervical pump delivery (39, 40).

### Limitations

The main limitation of this study is inherent to the fact that it's a case report and the results must be validated among a larger number of subjects before reaching any firm conclusions (41). But there are not many who can beneficiate from the method. We tried to present this work by taking the published recommendations (42–44). On the other hand, the team must have access to the necessary equipment which is widespread in the neurosurgical departments or in the hybrid room for interventional radiology (6, 7, 22).

## Conclusion

To our knowledge, it is the first time that a C1–2 lateral approach was described to cervical ITB spasticity treatment in a case of post traumatic SCI. But the method was proposed in other indications like cerebral palsy or in cancer pain treatment. We applied the protocol used to perform lateral cervical cordotomy with per operative O ‘arm guiding which is an efficient and safe technique.

This method is a reliable surgical technique with minimal risks, providing access to the cervical CSF when there is an obstacle in the catheter's path. Strict adherence to the preoperative assessment, procedural steps, and intraoperative imaging is essential to ensure optimal catheter placement.

The high cervical delivery of a drug might be a very interesting solution due to the important oscillatory flow at this level but there is a lack of fundamental works specifically studying this subject.

Further studies on the cervical tip localization and, more broadly, on a workflow management within this challenging population are warranted (34).

## Data Availability

The original contributions presented in the study are included in the article/Supplementary Material, further inquiries can be directed to the corresponding author.
